# Developmental relations between ADHD and self-esteem: evaluating peer problems as a mediating mechanism

**DOI:** 10.1007/s00787-025-02752-3

**Published:** 2025-05-31

**Authors:** Amanda Russell, Dejla Hoxha, Aja Murray

**Affiliations:** 1 Department of Psychology, Northumbria University, Newcastle upon Tyne, UK; 2 Department of Psychology, University of Edinburgh, Edinburgh, EH8 9JZ UK

**Keywords:** ADHD, Self-esteem, Peer problems, Adolescence

## Abstract

Previous research has suggested that adolescents with attention deficit hyperactivity disorder (ADHD) symptoms experience poorer self-esteem than their peers. Difficulties with peer problems may represent a key mediating mechanism. Using a large UK longitudinal study (the Millennium Cohort Study), we fit longitudinal mediation models (RI-CLPMs) across ages 11,14, and 17 (*n* = 4912 male, *n* = 4825 female) to examine the (bi-)directional relations among ADHD symptoms, peer problems, and self-esteem. We additionally examined the mediating role of peer problems in the developmental relation between ADHD symptoms and self-esteem. We found some evidence of reciprocal developmental associations between ADHD symptoms, peer problems, and self-esteem; however, peer problems did not mediate the links between ADHD symptoms and later self-esteem. Results suggest that there are inter-linkages between ADHD symptoms, peer problems and self-esteem that could be taken account of in interventions, such as addressing the self-esteem effects of peer problems in social skills interventions. Future research studies addressing the relations between ADHD symptoms, peer problems, and self-esteem over shorter time lags are also recommended.

Attention-deficit/hyperactivity disorder (ADHD) is characterised by a persistent pattern of severe inattention and/or hyperactivity/impulsivity [1]. The estimated global prevalence of ADHD in children and adolescents has varied across recent studies but is typically in the range of 5–8%, [2, 3, 4]. ADHD symptoms are, however, etiologically and phenotypically on a continuum, meaning that many are affected at subclinical levels [5, 6, 7]. By extension, examining the impact of ADHD symptoms in the general population is valuable for informing reductions in adverse outcomes associated with ADHD symptoms for those with and without a clinical diagnosis.

Despite potential protective characteristics associated with ADHD, such as the presence of positive illusory biases which may reduce awareness of difficulties [8, 9], low self-esteem is common in adolescents with ADHD symptoms [10, 11]. Low self-esteem has also been related to some of the negative outcomes associated with ADHD symptoms such as depression [12, 13]. It is, therefore, valuable to explore the mechanisms by which ADHD symptoms may engender lower self-esteem during this period to inform how to support better self-esteem. Whilst there are likely to be multiple pathways linking ADHD symptoms, the peer problems that are commonly associated with ADHD symptoms [14] may be a prominent pathway that is amenable to intervention. The purpose of this study is, therefore, to explore how, over the course of adolescence, ADHD symptoms, peer problems and self-esteem are developmentally related.

Growing evidence has suggested that ADHD and ADHD symptoms are related to developing impairments in self-esteem [10, 11, 12, 15]. A causal interpretation of this association is supported by the fact that self-esteem responds positively to ADHD treatment [15]. Illuminating the mechanisms underlying the association could help inform the prevention of self-esteem issues by identifying key targets for intervention lying on the pathway between ADHD symptoms and low self-esteem. Doing so is especially valuable during adolescence, which is a critical time of development with respect to self-esteem both in general populations and in those with ADHD or ADHD symptoms [16, 17].

There are likely to be multiple mechanisms by which ADHD symptoms engender self-esteem difficulties. These may include experiencing academic and psychosocial ‘failures’, for example, behavioural issues leading to sanctions, difficulties in family and social relationships, and academic difficulties [12, 18, 19]. However, a key candidate mechanism relates to the peer difficulties that are commonly associated with ADHD symptoms. Young people experiencing ADHD symptoms commonly face peer difficulties such as dislike, rejection, victimisation, poor friendship quality and bullying involvement [20, 21]. These are assumed to be due to ADHD-related difficulties such as social skills issues (e.g., difficulty with turn-taking due to impulsivity, missing social cues due to attention issues) or emotion dysregulation (e.g., being perceived as intense by peers leading to negative social evaluations). Reactive aggression is also common among young people with ADHD symptoms which can lead to outcomes such as rejection, or being perceived as a desirable target for victimisation for perpetrators who seek to solicit an ‘explosive’ reaction [14, 22]. Associations between ADHD symptoms and peer functioning have been found in relation to both ADHD diagnosis and ADHD symptoms levels [21, 23, 24, 25]. However, for those with a clinical diagnosis of ADHD, labelling may additionally carry stigma and an increased likelihood of rejection and poor peer functioning [26, 27].

Though peer difficulties can have a negative impact at any stage of development, difficulties in adolescence may have particular significance for self-esteem. This period of development is marked by a magnified importance of peers and of peer acceptance [28, 29]. Indeed, it has been argued that navigating peer relationships is a core developmental task of adolescence [30, 31] and a lack of ‘success’ in this domain may carry long-term negative consequences [32, 33].

A small number of studies have explored the role of peer difficulties in the links between ADHD/ ADHD symptoms and low self-esteem. These studies have found, for example, that adolescents with ADHD may view themselves negatively because of their perceived diminished social status when using social comparisons amongst peers [20]. However, there remains a need for studies that assess the long-term developmental relations between ADHD symptoms, peer problems and lower self-esteem. These studies should consider the potential reciprocal relations among these concepts, which would point to additional points of intervention to break the links between ADHD symptoms and poor outcomes. For example, low self-esteem could lead to social withdrawal, which would reduce opportunities for practising social skills and positive peer experiences as part of a perpetuating cycle.

It is also important to consider the role of sex and gender in the associations between ADHD symptoms, peer problems, and lower self-esteem. Here, ‘sex’ refers to biological/physiological characteristics while ‘gender’ captures socially constructed roles and attributes that society considers appropriate for different genders. It can be difficult to separate the effects of sex versus gender because socialisation of gender tends to begin early in life. Studies have previously shown that ADHD symptoms, peer problems and self-esteem can show sex and/or gender differences in terms of both levels and manifestation [34, 35]. This means that not considering sex/gender could in principle result in a confounding of the associations by acting as ‘common cause’ of all three domains. Moreover, there could be sex/gender differences in the relations between ADHD symptoms, peer problems and self-esteem. For example, there may be elevated vulnerability for adverse developmental links by sex/gender (e.g., if the links between ADHD symptoms, peer problems and self-esteem are stronger in females due to a greater sensitivity to the effects of peer difficulties) [36]. Similarly, some research suggests that ADHD symptoms are less related to peer problems in boys than in girls because peers tolerate higher levels of ADHD symptoms in boys where they are perceived to be more gender appropriate [37]. There has been little research on sex/gender differences in these linkages to date. One study examined trajectories of self-worth – a closely related concept to self-esteem - in adolescents with ADHD, finding no evidence for sex differences [38]. Another found that relational victimisation as a form of peer problems was associated with poorer self-esteem in boys but not girls with ADHD [39]. However, specific research on sex/gender differences in the linkages among ADHD symptoms, peer problems and self-esteem remains scarce.

It is also important that studies disentangle the between- and within-person associations among these concepts as it is the latter that is most informative in regards to potential intervention targets [40]. Specifically, between-person associations reflect associations between stable trait-like components that differ between people whereas within-person associations reflect how one outcome (e.g., self-esteem) improves or declines as individuals’ increase or decrease on another (e.g., peer problems). Though this is not always made explicit, it is these latter relations that are typically of interest when designing and implementing interventions. The cross-lagged panel model (CLPM) widely used to examine developmental relations over time provides parameters that are a mixture of between and within-person effects. This can result in misleading conclusions, for example, when the parameters are interpreted as within-person effects but are in reality driven by between-person confounds [41]. Fortunately, recent advances facilitate extensions of the model such as the random intercept CLPM (RI-CLPM) that allow these effects to be separated and the within-person effects of interest to be isolated from between-person confounds [40, 42].

Given the value in illuminating the pathways that result in lower self-esteem among adolescents with ADHD and the evidence pointing to peer problems as a key plausible mechanism, we here evaluated the developmental relations among ADHD symptoms, peer problems, and self-esteem in a large UK-based longitudinal study. Relations were evaluated across ages 11,14, and 17 and the mediating role of peer problems in the link between ADHD symptoms and later self-esteem was tested. We hypothesised that higher ADHD symptoms would be related to higher within-person peer problems and lower self-esteem over development and that the latter association would be mediated by peer problems. We hypothesised that these associations would be present in both males and females.

## Methods

### Participants

Participants were from the waves of the Millennium Cohort Study (MCS) where they were aged d ~11,14, and 17. MCS is a large UK-based longitudinal birth cohort study, with over 18,000 children enrolled at baseline [43]. MCS began data collection when the participants were aged 9 months and were eligible based on being born in the UK between September 2000 and January 2002. Families living in deprived areas and wards with high ethnic minority populations were oversampled to ensure adequate representation of these groups. Ethical approvals were granted and informed consent obtained for all sweeps of data collection.

We include all participants with available data from MCS in the current analysis irrespective of ADHD diagnosis because our goal is to understand the effects of ADHD symptoms, rather than a categorical classification of whether a person meets or does not meet diagnostic criteria for ADHD. There were 10,078 participants included in our analysis based on having remained in the study and contributed data up to age 17 (*n* = 4825 females and *n* = 4912 males) of whom 109 and 131 had a parent-reported ADHD diagnosis at age 11 and 14 respectively. In terms of the ethnic composition of the sample, most participants were White (*n* = 8021), *n* = 274 were classified as Mixed, *n* = 263 were classified as Indian, *n* = 726 were classified as Pakistani and Bangladeshi, *n* = 301 were classified as Black or Black British, and *n* = 136 were classified as Other Ethnic Group. In terms of socioeconomic status, the modal total joint annual income of the households in the sample was in the ‘from 40,500 and less than 48,000’ GBP bracket at the age 11 wave of data collection.

## Measures

### ADHD symptoms

ADHD symptoms were measured using the parent-reported Strengths and Difficulties Questionnaire (SDQ) hyperactivity/inattention subscale [44]. Despite some identified limitations, the SDQ is a widely used and well-validated brief behavioural screening instrument [45], with the hyperactivity/inattention subscale previously shown to be a good predictor of ADHD diagnosis [46]. In the present sample it has shown evidence of good internal consistency reliability, as well developmental and cross-informant invariance up to a high level [47, 48]. Specifically, previous research has found that the SDQ shows gender measurement invariance up to the residual level across ages 5 to 14; longitudinal invariance up to the partial residual level across ages 5 to 14; and teacher-parent informant invariance at age 11 up to the scalar level [47, 48].

The hyperactivity/inattention subscale comprises 5 items, asking parents about their child’s behaviour over the last 6 months. Items relate to: ‘restlessness, overactive, cannot stay still for long’, ‘constantly fidgeting or squirming’, ‘easily distracted, concentration wanders’, ‘thinks things out before acting’, ‘sees tasks through to the end, good attention span.’ Items are rated on a 3-point Likert scale: *Not true (0)*,* Somewhat true (1)* and *Certainly true (2)*. Respondents are also offered an additional response option of ‘Can’t Say’. A composite score was used in the present study, created by summing the individual item scores. The composite score has a possible range of 0–10, with higher scores representing higher levels of ADHD symptoms. Omega reliability for the score in the current sample was 0.82, 0.85, and 0.80 at ages 11, 14, and 17 respectively indicating good internal consistency reliability.

### Peer problems

Peer problems were measured through the peer problems subscale of the SDQ. Parents answered 5 items: ‘rather solitary tends to play alone’, ‘has at least one good friend’, ‘generally liked by other children’, ‘picked on or bullied by other children’, ‘gets on better with adults than with other children’. Items were rated using the 3-point Likert scale as described above. A composite score was used in the current study, created by summing the individual item scores. These scores have a possible range from 0 to 10, with higher scores representing higher levels of peer problems. Omega reliability for the score in the current sample was 0.73, 0.78 and 0.72 at ages 11, 14, and 17 respectively indicating good internal consistency reliability.

### Self-esteem

Participants’ self-esteem was assessed through self-reports, using the five positively worded items of Rosenberg self-esteem scale (RSES; Rosenberg, 1979) available in the dataset. RSES is a well-validated global self-esteem measurement tool that has been widely used among those with ADHD. It includes items such as “I feel that I am a person of worth” and “I feel good about myself”. It uses a 4-point Likert scale from *Strongly agree* to *Strongly disagree*. ‘Don’t know’ and ‘I do not wish to answer’ options are also provided. All items are worded ‘positively’, meaning that higher scores (indicating stronger disagreement) represent worse self-esteem. A total score was derived by summation of individual item scores, giving a possible range from 0 to 20 (with higher scores representing worse self-esteem). In the current sample it has shown good psychometric properties, including high internal consistency values [50]. Omega reliability at ages 11, 14 and 17 was 0.83, 0.94, and 0.95, indicating good internal consistency reliability.

### Statistical procedure

A random-intercept cross-lagged panel model (RI-CLPM) was fit to the data. This model can disaggregate between- and within-person associations. It does so by specifying random intercept factors for each concept (ADHD symptoms, peer problems, self-esteem) which are allowed to covary at the between person-level. It then fits a cross-lagged structure to the time-specific residuals for each concept.

The between-person covariances capture associations in average levels of (or individual differences in) each concept across people (e.g., whether those higher in ADHD symptoms tend to also have lower self-esteem and higher peer problems).

The within-person cross-lagged structure involves both lag-1 autoregressions (each concept predicts itself at the next wave) and lag-1 cross-lagged paths (each concept predicts each other concept at the next wave). These model how ‘changes’ in individuals’ levels of these three concepts over time affect their levels at the next time point. More precisely, they assess how an individual’s deviation from their average level of a concept (e.g., ADHD symptoms) relates to their deviation from their average level of the same and other concepts (e.g., peer problems, self-esteem) at the next time point. This exploits the fact that we know that young people’s ADHD symptoms can fluctuate and/or systematically increase or decrease over development [50, 51, 52]. Conceptually, it assesses whether escalations in peer problems follow escalations in ADHD symptoms, and whether this is, in turn, associated with later decrements in self-esteem. As each individual is compared to themselves at other time-points, it helps adjust for confounding factors that vary between people but are stable over time. In addition, within-time covariances are included between the residuals at each time point to capture concurrent within-person associations.

To assess mediation of the association between ADHD symptoms and self-esteem by peer problems, the product of the coefficients for the regression of self-esteem at age 17 on peer problems at age 14 and the regression of peer problems at age 14 on ADHD symptoms at age 11 was formed. The statistical significance of this coefficient was assessed using bootstrapped 95% confidence intervals. Models were fit in Mplus using pseudo-maximum likelihood (PML) design-adjusted analyses to take account of the complex survey design [53].

To reduce the complexity of the model we used composite scores (rather than latent variable models) for each construct, formed as the sum of the item scores for each construct at age wave. Whole sample, as well as sex-stratified analyses were conducted since previous studies have suggested sex differences in ADHD symptoms, peer problems, and self-esteem [39, 51, 54].

## Results

### Descriptive statistics

Descriptive statistics are provided in Table 1.

**Table 1:** Descriptive statistics for ADHD symptoms, peer problems and Self-Esteem at ages 11,14 and 17

Variable	*N*	Mean	SD	Min	Max
Age 11 ADHD symptoms	12,969	9.19	1.59	5	20
Age 14 ADHD symptoms	11,055	9.00	1.43	5	15
Age 17 ADHD symptoms	9774	10.03	1.56	5	15
Age 11 Peer problems	12,969	9.92	1.34	5	20
Age 14 Peer problems	11,021	9.97	1.27	5	15
Age 17 Peer problems	9781	9.75	1.21	5	15
Age 11 Self-esteem	12,239	8.04	2.17	5	20
Age 14 Self-esteem	11,141	9.43	2.89	5	20
Age 17 Self-esteem	10,052	9.98	3.19	5	20

*Note*. SD = standard deviation. Descriptive statistics are unweighted

These suggest fairly similar levels of peer problems over adolescence but slight increases in ADHD symptoms and worse self-esteem issues at age 17. The inter-correlations of the scores (Pearson correlations) are provided in Table 2. All correlations were significant at *p* < .05 except between ADHD symptoms at age 11 and self-esteem at ages 11,14 and 17 and between self-esteem and ADHD symptoms at age 14.

**Table 2:** Inter-Correlations among ADHD symptoms, peer problems, and Self-Esteem at ages 11,14 and 17

	1.	2.	3.	4.	5.	6.	7.	8.	9.
1. ADHD age 11									
2. ADHD age14	0.38*								
3. ADHD age 17	0.08*	0.11*							
4. Peer problems age 11	0.28*	0.12*	0.04*						
5. Peer problems age 14	0.12*	0.16*	0.05*	0.33*					
6. Peer problem age 17	0.05*	0.03*	0.17*	0.11*	0.15*				
7. SE age 11	0.00	0.00	0.04*	0.08*	0.09*	0.03*			
8. SE age 14	− 0.01	− 0.03*	0.06*	0.05*	0.08*	0.04*	0.29*		
9. SE age 17	− 0.02	− 0.05*	0.10*	0.04*	0.07*	0.06*	0.19*	0.39	

Note. *= significant at *p* < .05. ADHD = Attention deficit hyperactivity disorder symptoms; SE = Self-esteem

### RI-CLPM

Key parameters from the RI-CLPM fit to the whole sample are provided in Table 3 with full results provided at: https://osf.io/gfhvj/files/osfstorage. These results suggest significant within-person cross-lagged effects of ADHD symptoms at age 14 on later peer problems and lower self-esteem at age 17 but across no other lag. There were significant effects of peer problems on later lower self-esteem across both lags. There was some evidence of reciprocal effects, with lower self-esteem additionally impacting peer problems across the age 11 to 14 lag and impacting ADHD symptoms across both lags. There was, however, no significant indirect effect of ADHD symptoms on later self-esteem via peer problems (indirect effect: b = 0.002, 95% CI = -0.002, 0.008). The ADHD symptoms random intercept factor was significantly associated with the self-esteem (estimate = 0.160, *p* = .003) and peer problems random intercept factors (estimate = 0.116, *p* = .002), suggesting that ADHD symptoms are positively associated with both peer problems and lower self-esteem. The peer problems and self-esteem random intercepts factors were not significantly associated.

Sex-stratified RI-CLPM results are provided in Table 3. In the male sample the within-person results were highly similar to those in the whole sample, though fewer parameters were significant, likely due to the reduced sample size. One parameter: the effect of peer problems at age 11 on lower self-esteem at age 14 was significant in males but not the whole sample. In common with the whole sample analyses, there was no significant indirect effect (b = 0.002, 95% CI = -0.007, 0.014). However, unlike the whole sample analyses, the only significant between-person association was the ADHD and peer problems random intercept factor covariance (estimate = 0.106, *p* = .019).

In the female sub-sample analyses, within-person results were also similar to the whole sample analyses albeit with fewer significant parameters overall, again likely due to the reduced sample size. There was no significant indirect effect (b = 0.003, 95% CI = -0.003, 0.014) and there were no significant between-person (random intercept factor) associations in the female sub-sample.

**Table 3:** Key RI-CLPM Within-Person parameters (Unstandardised)

		Whole sample (*N* = 10078)					Males (*N* = 4912)				Females (*N* = 4825)		
		Estimate	SE	*p*			Estimate	SE	*p*		Estimate	SE	*p*
	Within-person regressions												
Self-esteem age 17 regressed on…													
Self-esteem age 14		0.348	0.026	< 0.001			0.357	0.034	< 0.001		0.278	0.052	< 0.001
Peer problems age 14		0.132	0.043	0.002			0.168	0.059	0.004		0.105	0.069	0.125
ADHD symptoms age 14		-0.142	0.037	< 0.001			-0.111	0.050	0.028		-0.143	0.060	0.017
Peer problems age 17 regressed on…													
Self-esteem age 14		-0.008	0.013	0.523			-0.013	0.020	0.513		-0.01	0.018	0.583
Peer problems age 14		0.083	0.035	0.016			0.109	0.039	0.005		0.056	0.058	0.340
ADHD symptoms age 14		-0.052	0.022	0.016			-0.056	0.031	0.071		-0.035	0.031	0.250
ADHD symptoms age 17 regressed on…													
Self-esteem age 14		0.031	0.012	0.009			0.065	0.018	< 0.001		-0.008	0.019	0.683
Peer problems age 14		-0.005	0.031	0.874			0.001	0.041	0.977		-0.01	0.046	0.833
ADHD symptoms age 14		0.032	0.029	0.267			0.048	0.041	0.242		0.027	0.042	0.528
Self-esteem age 14 regressed on…													
Self-esteem age 11		0.249	0.046	< 0.001			0.206	0.063	0.001		0.236	0.063	< 0.001
Peer problems age 11		0.065	0.052	0.215			0.214	0.069	0.002		-0.022	0.071	0.757
ADHD symptoms age 11		-0.063	0.039	0.108			0.008	0.068	0.908		-0.068	0.055	0.216
Peer problems age 14 regressed on…													
Self-esteem age 11		0.041	0.015	0.005			0.050	0.023	0.031		0.041	0.019	0.031
Peer problems age 11		0.272	0.027	< 0.001			0.267	0.037	< 0.001		0.271	0.042	< 0.001
ADHD symptoms age 11		0.014	0.018	0.433			0.012	0.028	0.667		0.026	0.022	0.243
ADHD symptoms age 14 regressed on…													
Self-esteem age 11		-0.031	0.013	0.018			-0.036	0.021	0.078		-0.021	0.019	0.282
Peer problems age 11		-0.003	0.022	0.891			-0.040	0.031	0.186		0.029	0.026	0.276
ADHD symptoms age 11		0.299	0.020	< 0.001			0.301	0.031	< 0.001		0.291	0.027	< 0.001
	Within-person concurrent associations												
Self-esteem age 17 with…													
Peer problems age 17		0.062	0.135	0.643			-0.109	0.238	0.647		0.212	0.118	0.072
ADHD age 17		0.411	0.079	< 0.001			0.593	0.126	< 0.001		0.185	0.091	0.041
Peer problems age 17 with…													
ADHD age 17		0.148	0.064	0.021			0.089	0.105	0.395		0.249	0.055	< 0.001
Self-esteem age 14 with…													
Peer problems age 14		0.220	0.063	< 0.001			0.22	0.086	0.011		0.216	0.086	0.012
ADHD age 14		-0.162	0.06	0.007			-0.111	0.084	0.185		-0.107	0.083	0.196
Peer problems age 14 with…													
ADHD age 14		0.165	0.036	< 0.001			0.123	0.04	0.002		0.205	0.05	< 0.001
Self-esteem age 11 with…													
Peer problems age 11		0.278	0.084	0.001			0.221	0.086	0.010		0.368	0.138	0.008
ADHD age 11		-0.065	0.065	0.319			-0.043	0.102	0.675		-0.086	0.102	0.399
Peer problems age 11 with…													
ADHD11		0.381	0.049	< 0.001			0.329	0.067	< 0.001		0.431	0.075	

Note. SE = Standard Error. ADHD = attention deficit hyperactivity disorder symptoms

## Discussion

Previous research has suggested that there may be potential developmental pathways between ADHD symptoms and self-esteem in adolescence via peer problems. Illuminating these pathways could identify potential intervention points to support better self-esteem for this group. In a large longitudinal study, we found evidence of reciprocal developmental relations among ADHD symptoms, peer problems, and lower self-esteem. However, there were no significant mediating effects of peer problems in the relation between early adolescent ADHD symptoms and later self-esteem issues. As such, our hypotheses were only partially supported.

Results are consistent with several previous studies that have identified links among ADHD symptoms, peer problems and lower self-esteem [10, 11, 12, 13, 15, 39, 55, 56]. However, our study extends these findings by evaluating developmental links in a rigorous longitudinal data analysis that disaggregated between- and within-person effects and by explicitly testing mediation. This method provides more relevant information to inform interventions than traditional CLPMs because it is these within-person effects that are typically – implicitly or explicitly – the targets of intervention [40].

Our findings suggested potential reciprocal effects among ADHD symptoms, peer problems and lower self-esteem over time. Specifically, as well as ADHD symptoms impacting later peer problems (at age 14 and 17) and lower self-esteem (at age 14), ADHD symptoms were impacted by earlier lower self-esteem (at ages 11 and 14). The impact of lower self-esteem on ADHD symptoms may be a reflection of the negative effects of self-esteem issues on outcomes such as anxiety and depression and associated issues such as worry and agitated mood. This could result in the consumption of attentional resources and potentially exacerbate restlessness [57]. Self-esteem and peer problems also showed some evidence of reciprocal associations. Self-esteem effects on peer problems could reflect potential impacts of low self-esteem on rejection sensitivity [58] or social withdrawal impacting peer functioning. However, further research will be required to establish the mechanisms underlying these reciprocal associations.

These associations underline that self-esteem and peer problems are important targets for intervention among adolescents experiencing ADHD symptoms. Their reciprocal nature also implies multiple entry-points into improving functioning for adolescents with ADHD symptoms and suggests that combined approaches could be helpful. For example, based on the link between self-esteem and later peer functioning, interventions to improve peer functioning for those with ADHD or ADHD symptoms (see e.g., Morris et al., 2021) could incorporate elements aimed at bolstering self-esteem and mitigating the impacts of low self-esteem on social functioning. A variety of intervention approaches have been trialled to target improvements in self-esteem, with cognitive and behavioural approaches such as cognitive behavioural therapy (CBT), arguably best-evidenced in adults [60]. However, although some interventions have been proposed and evaluations conducted, the evidence is considerably more limited with respect to interventions to improve self-esteem that are specifically tailored to adolescents with ADHD symptoms [61, 62, 63]. Overall, there remains a need for theoretically and empirically informed interventions to target those experiencing low self-esteem, tailored to their specific needs [64], especially those that take account of the inter-connections between ADHD symptoms, peer problems, and self-esteem. The findings also point to a potential knock-on benefit of successfully identifying and treating ADHD symptoms through, for example, pharmacotherapy or psychosocial interventions [65] ; namely, the downstream reduction in peer and self-esteem issues.

Despite the presence of developmental associations among ADHD symptoms, peer problems and self-esteem, there were no significant mediating effects. Specifically, effects of age 11 ADHD symptoms on age 17 self-esteem were not mediated by age 14 peer problems. The lack of effect may reflect the long time lag over which these symptoms were measured. Indeed, it is unclear what the time-course of any mediating effects should be, making it difficult to establish a measurement design that has the optimal time lag to capture these effects [66]. It would thus be valuable to examine these associations over different timescales, for example, using ecological momentary assessment (EMA) designs to capture moment-to-moment processes [67] and shorter interval longitudinal studies to capture medium-term associations. The presence of several significant within-person concurrent associations also pointed to the use of shorter time lags. A further factor in the lack of mediating effect may be that the links between ADHD symptoms and self-esteem may be mediated by many pathways of small effect.

### Future directions

There are a number of future directions suggested by our findings. First, following the identification of general associations between ADHD symptoms, peer problems and self-esteem, it would be valuable to employ more comprehensive multi-dimensional measures of each concept, as previous research has suggested that all three domains comprise multiple subdimensions. It would thus be helpful to delineate more specific associations to establish whether specific aspects are particularly inter-related. Previous research has suggested some nuances, for example, indicating that different ADHD symptoms may be differentially linked to different peer problems, such as withdrawal (inattention) versus rejection and reactive aggression (hyperactivity/impulsivity) [22]. It would also be valuable to examine the effects of peer problems alongside other mechanisms that may link ADHD symptoms to later self-esteem issues, such as behavioural issues and associated sanctions, family difficulties, and academic challenges to provide a more comprehensive picture and compare the relative importance of each potential pathway. Finally, it would be valuable to examine the inter-connections of ADHD symptoms, peer problems and self-esteem over a longer developmental span that traces their co-development from childhood through adolescence to adulthood. This would help establish optimal points of development at which to intervene, complementing what is already known about adolescence as a critical period for peer problems and self-esteem.

### Limitations

It is important to note the limitations of the present study. First, we relied on a single informant for all concepts whereas a multi-informant approach is the gold standard in the assessment of child and adolescent psychosocial outcomes, especially ADHD symptoms whereby situational consistency is an important aspect of diagnosis [68, 69]. Further, while we combined self-reports for self-esteem with parent reports for ADHD symptoms and peer problems, the use of the same informant for the latter two concepts may have resulted in their association being inflated due to common rater bias [70]. Further, as young people with elevated ADHD symptoms may show positive illusory biases (i.e., an underestimation of their difficulties) [8], it is possible that the self-esteem self-reports were overly optimistic for those with higher levels of ADHD symptoms. However, some evidence has suggested that self-concept, which is closely related to self-esteem may be unaffected by the presence of positive illusory bias [9 and others have noted that positive illusory biases may be present only for a minority of adolescents with ADHD [71]. As such, whether or not this is likely to be an important source of bias is unclear. Finally, it is important to note that because we focused on measuring ADHD symptoms based on a continuous measure of symptom levels our inferences are limited to the effects of ADHD symptoms rather than the effects of ADHD diagnosis as a categorical variable. It would be valuable to conceptually replicate the study in terms of examining the impact of ADHD as measured according to diagnosis (or meeting diagnostic criteria). Similarly, it would be valuable to examine whether the findings replicate within the population of adolescents with ADHD,

## Conclusions

Our findings suggest that there are reciprocal developmental linkages between ADHD symptoms, peer problems, and self-esteem across adolescence. However, we did not find evidence that peer problems mediated the effect of ADHD symptoms at age 11 on self-esteem at age 17. Results point to the importance of self-esteem and peer problems as intervention targets in adolescents with ADHD symptoms and suggest potential value in combined approaches that target both domains (and their inter-connections) simultaneously.

## Data Availability

Data are available via UK Data Service.
